# Association of Overweight, Obesity, and Recent Weight Loss With Colorectal Cancer Risk

**DOI:** 10.1001/jamanetworkopen.2023.9556

**Published:** 2023-04-21

**Authors:** Marko Mandic, Fatemeh Safizadeh, Tobias Niedermaier, Michael Hoffmeister, Hermann Brenner

**Affiliations:** 1 Division of Clinical Epidemiology and Aging Research, German Cancer Research Center (DKFZ), Heidelberg, Germany; 2 Institute for Medical Information Processing, Biometry and Epidemiology, Ludwig Maximilian University of Munich, Munich, Germany; 3 Medical Faculty Heidelberg, Heidelberg University, Heidelberg, Germany; 4 Division of Preventive Oncology, DKFZ and National Center for Tumor Diseases, Heidelberg, Germany; 5 German Cancer Consortium, DKFZ, Heidelberg, Germany

## Abstract

**Importance:**

Overweight and obesity, conditions with rising prevalence in many countries, are associated with increased colorectal cancer (CRC) risk. However, many patients with CRC lose weight before diagnosis, which may lead the association to be underestimated.

**Objective:**

To evaluate the association of body mass index (BMI) and weight change with CRC risk when considering BMI at different time frames, with the intention to account for prediagnostic weight loss.

**Design, Setting, and Participants:**

This population-based case-control study was conducted in southwestern Germany between 2003 and 2021. Cases with a first diagnosis of CRC and controls (frequency matched by age, sex, and county) with comprehensive risk factor information and self-reported weight at different time points were included. Data were analyzed between October 2022 and March 2023.

**Exposure:**

BMI and weight change at different time frames before the time of diagnosis (cases) or recruitment (controls).

**Main Outcomes and Measures:**

Association of BMI and weight change at various points in time before and up to diagnosis with CRC, assessed by multivariable logistic regression with comprehensive confounder adjustment.

**Results:**

A total of 11 887 participants (6434 CRC cases, 5453 controls; median [IQR] age, 69 [61-77] years; 7173 male [60.3%]) were included. At the time of diagnosis, 3998 cases (62.1%) and 3601 controls (66.0%) were overweight or obese, suggesting an inverse association between excess weight and CRC risk. Conversely, we found significant positive associations of overweight (adjusted odds ratio [aOR], 1.27; 95% CI, 1.03-1.56), obesity (aOR, 2.09; 95% CI, 1.61-2.70), and a 5-unit increase in BMI (aOR, 1.35; 95% CI, 1.21-1.50) with CRC risk when using BMI measured 8 to 10 years before diagnosis. High BMI as a risk factor for CRC was increased as earlier periods before diagnosis were examined, with the association being particularly pronounced using BMI at least 8 years before diagnosis. An opposite trend was found for the association of weight loss (at or exceeding 2 kg) with CRC, with the greatest effect sizes occurring for weight loss within 2 years before diagnosis (aOR, 7.52; 95% CI, 5.61-10.09), and gradually decreased for earlier intervals.

**Conclusions and Relevance:**

In this population-based case-control study, accounting for substantial prediagnostic weight loss further highlighted the association of overweight and obesity with CRC risk.

## Key Points

**Question:**

How does weight loss before diagnosis, which is common among patients with colorectal cancer (CRC), affect the association of overweight and obesity with risk of CRC?

**Findings:**

In this case-control study, an inverse association was observed between overweight and CRC risk when body mass index (BMI) at the time of recruitment was considered. However, recent weight loss was associated with strongly increased CRC risk, and a clear positive association between BMI and CRC risk emerged when looking at BMI at earlier points of time, which was particularly pronounced for BMI 8 or more years ago.

**Meaning:**

These findings suggest that prediagnostic weight loss bias can lead to a considerable underestimation of the association of BMI with CRC.

## Teaser

This case-control study examines how recent weight loss of patients with colorectal cancer may affect estimates of the association of colorectal cancer risk with overweight or obesity.

## Introduction

Excess body weight is an established risk factor for a variety of cancers, including colorectal cancer (CRC).^1^ Prevalence of overweight and obesity, which are commonly defined by a body mass index (BMI; calculated as weight in kilograms divided by height in meters squared) from 25 to 30 and 30 or above, respectively, has been steadily rising in many countries.^2,3^ According to the World Health Organization (WHO), overweight and obesity affected more than one-third of the global population in 2016, with around 2 billion adults and nearly 400 million adolescents and children being overweight or obese.^4^ Recent systematic reviews suggest that individuals with obesity have about 30% greater risk of CRC compared with those with normal weight.^5,6,7^ However, excess body weight as a risk factor for CRC might have been underestimated in previous epidemiological studies due to an often-overlooked source of bias—prediagnostic weight loss. Mean sojourn time in the preclinical state has been estimated to be around 3 to 6 years for CRC.^8,9,10^ This prediagnostic period often goes along with weight loss.^11,12^ Most studies have ascertained body weight at a single point in time, usually close to the time of cancer diagnosis (case-control studies) or participant enrollment (cohort studies), ie, at the time when cancer-associated weight loss may already have occurred. Furthermore, in a 2023 umbrella review,^13^ we have shown that none of the previous 18 reviews looking at the BMI-CRC association considered prediagnostic weight loss as a potential source of bias. This may have led to an underestimation of the association of overweight and obesity with CRC risk in epidemiological studies, but the magnitude of such underestimation is unknown.

This study aimed to assess how prediagnostic weight loss may affect the association of excess weight with CRC risk. Using data from a large population-based case-control study, we analyzed the association of BMI at various points in time before CRC diagnosis with CRC risk, with a special focus on the role of potential weight changes in the 12-year window before cancer diagnosis.

## Methods

### Study Design and Study Population

Our analysis was based on data from the DACHS study (Darmkrebs: Chancen der Verhütung durch Screening), a population-based case-control study conducted in southwestern Germany between 2003 and 2021. Details of the study have been reported elsewhere.^14,15^ Briefly, patients with a histologically confirmed first diagnosis of CRC (*International Statistical Classification of Diseases and Related Health Problems, Tenth Revision* [*ICD-10*] codes C18-C20) were eligible to participate if they were at least 30 years of age, spoke German, and were physically and mentally able to participate in an interview of about 1 hour. No upper age limit was used. Community-based controls were randomly selected from population-based registries, using frequency matching for age, sex, and county of residence. Controls with a history of CRC were excluded; otherwise, inclusion and exclusion criteria were the same as for cases.

The study was approved by the ethics committees of the Heidelberg Medical Faculty of Heidelberg University and the state medical boards of Baden-Württemberg and Rhineland-Palatinate. Our analysis was based on cases and controls who were recruited between 2003 and 2019. Written informed consent was obtained from each participant. This study followed the Strengthening the Reporting of Observational Studies in Epidemiology (STROBE) reporting guideline.

### Data Collection

Patients were informed about the study by clinicians, usually during or shortly after their hospital stay for CRC surgery. All of the clinics providing CRC surgery in the catchment area of approximately 2 million people (more than 20 clinics) contributed to recruitment. Patients participated in an interview with trained interviewers who collected information on patients’ sociodemographic, medical, and lifestyle history using a standardized questionnaire. Patients who could not be recruited during their hospital stay were contacted by mail shortly after discharge by clinicians or clinical cancer registries, and interviews were conducted at their homes. According to estimates based on cancer registries, approximately 50% of eligible cases in the study area could be recruited. Controls were contacted by the study centers through mail and follow-up calls, and interviews using the same standardized questionnaire were scheduled at their homes (participation rate was 51%). A minority of control participants, who were not willing to participate in a personal interview but provided key information in a self-administered questionnaire instead, were excluded from this analysis because information on BMI at different ages was not obtained from those participants. Information on weight at preceding ages (measured at 10-year intervals at age 20 years through age 80 years) and current weight and height were obtained from self-reports during the interview.^16^ Participants older than 90 years did not provide information about weight at age 90 years and were excluded from the analysis.

### Statistical Analysis

Demographic and lifestyle characteristics of cases and controls are presented using descriptive statistics, and their distribution was compared by Pearson χ^2^ test. For all covariates with missing values, we performed multiple imputations. All further analyses were conducted with the 5 imputed data sets and the results of each individual data set were combined. Multiple imputation procedures were performed in R version 4.2.2 (R Project for Statistical Computing) using the mice package.^17^ Matching factors (age, sex, and county), height, weight at each decennial age, and potential confounders were included in the imputation procedure.

To assess how avoiding the period of potential prediagnostic weight loss affects the BMI-CRC association, we considered information on weight and weight changes at various time windows within 12 years before diagnosis (cases) or interview (controls). Due to the way information on previous weight was collected (ie, weight at age 20, 30, 40 years, etc), weights in specific time windows before diagnosis or interview were available from defined subgroups of the study population only (for each preceding year approximately 10% of cases and controls) (eTable 1 in the Supplement 1). For example, information on weight 1 to 2 years ago, ie, around participants’ decennial birthdays, was available from cases and controls aged 31, 41, 51, 61, 71, and 81 years at diagnosis or interview only. Changes in reported weight between various 2-year intervals before diagnosis or interview (0 to <2, 2 to <4, 4 to <6, 6 to <8, 8 to <10, and 10 to <12 years ago) and current weight were visualized for cases and controls by box-whisker plots.

Multivariable logistic regression was used to estimate odds ratios (ORs) and their 95% CIs for the association of BMI and CRC. Two adjustment levels were applied. Model 1 adjusted for age and sex. Model 2 (main results) additionally adjusted for level of education, smoking, alcohol consumption, physical activity, family history of CRC, previous lower gastrointestinal endoscopy, and use of nonsteroidal anti-inflammatory drugs (NSAIDs) and statins. Four categories (according to the WHO definition)^2^ of BMI (underweight, below 18.5; normal weight, 18.5 to below 25; overweight, 25 to below 30; and obesity, 30 or higher) were used. Additionally, we estimated ORs per 5-unit increase in BMI using BMI as a continuous variable.

To evaluate associations between BMI at different time frames before diagnosis or interview and CRC, we carried out the following analyses: we first determined the association of BMI based on current weight with CRC risk. Next, we replaced current weight by weight within various past 2-year intervals (0 to 2 years ago through 10 to 12 years ago) (eTable 2 in the Supplement 1). Again, to quantify the risk of CRC per each BMI category and per 5-unit increase in BMI, we used logistic regression models adjusting for the matching variables (model 1) and models with comprehensive confounder adjustment (model 2). In addition, for each time frame, we assessed the association of weight change (categorized as weight loss of 2 kg or more, weight gain of 2 kg or more, and no change) and CRC risk. We performed subgroup analyses by sex and cancer site (colon, rectum), and we additionally evaluated the association of weight change defined as a 5% or greater body weight change with CRC risk. All statistical analyses were conducted using R version 4.2.2. Statistical tests were 2-sided, and *P* < .05 was considered statistically significant for all tests.

## Results

### Characteristics of the Study Participants

**Figure 1.**
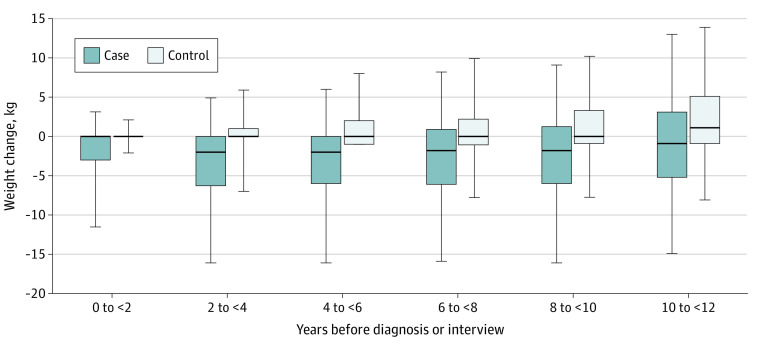
Weight Change Among Cases and Controls Within 12 Years Before Diagnosis/Interview Lower and upper hinges correspond to 25th and 75th quantile of weight changes, respectively; lower and upper whiskers correspond to 5th and 95th quantile, respectively; outliers not shown.

A total of 11 887 DACHS participants (6434 cases, 5453 controls) were included in the analysis (eFigure in Supplement 1). Cases had a median (IQR) age of 69 (61-77) years, and 3857 (60.0%) were male; controls had a median age of 69 (61-77) years, and 3316 (60.8%) were male (Table 1). Cases more often had a low level of education (9 years or less: 4106 participants [63.8%] vs 2780 [51.0%]), were more likely to have a family history (first-degree) of CRC (935 [14.5%] vs 610 [11.2%]), to be current smokers (1022 [15.9%] vs 631 [11.6%]), to drink more alcohol (more than 21.8 g/d: 1561 [24.2%] vs 1157 [21.2%]), to have diabetes (1232 [19.1%] vs 714 [13.1%]), to have had lower gastrointestinal endoscopy less often (1788 [27.8%] vs 3395 [62.2%]), and were less likely to use NSAIDs (1595 [24.7%] vs 1648 [30.5%]) or statins (1190 [18.5%] vs 1214 [22.3%]) than controls. Figure 1 shows the distribution of weight changes in cases and controls since different 2-year intervals up to 12 years before diagnosis or interview. Whereas the majority of controls had increased their weight since these past time intervals, the opposite was the case for the majority of cases.

**Table 1.** Characteristics of the Study Participants

Characteristics	No. (%) (N = 11 887)		P value
	Cases (n = 6434)	Controls (n = 5453)	
Sex			
Female	2577 (40.0)	2137 (39.2)	NA
Male	3857 (60.0)	3316 (60.8)	
Age, y			
Median (IQR)	69 (61-77)	69 (61-77)	NA
Education, y			
≤9	4106 (63.8)	2780 (51.0)	<.001
10-11	1153 (17.9)	1237 (22.7)	
12-13	1175 (18.3)	1436 (26.3)	
Smoking status^a^			
Never	2862 (44.5)	2760 (50.6)	<.001
Former	2550 (39.6)	2062 (37.8)	
Current	1022 (15.9)	631 (11.6)	
Alcohol consumption, g/d^b^			
Never	1173 (18.2)	809 (14.8)	<.001
<4.7	1272 (19.7)	1140 (20.9)	
4.7-10.6	1188 (18.5)	1174 (21.5)	
10.6-21.8	1240 (19.3)	1173 (21.5)	
>21.8	1561 (24.2)	1157 (21.2)	
Physical activity, MET-h/wk^c^			
<119.5	1461 (22.7)	1362 (25.0)	<.001
119.5-175.6	1546 (24.0)	1363 (25.0)	
175.6-242.1	1620 (25.2)	1364 (25.0)	
>242.1	1807 (28.1)	1364 (25.0)	
History of CRC in first-degree relative			
No	5499 (85.5)	4843 (88.8)	<.001
Yes	935 (14.5)	610 (11.2)	
Previous lower gastrointestinal endoscopy			
No	4646 (72.2)	2058 (37.8)	<.001
Yes	1788 (27.8)	3395 (62.2)	
Diabetes			
No	5202 (80.9)	4739 (86.9)	<.001
Yes	1232 (19.1)	714 (13.1)	
NSAIDs use^d^			
No	4839 (75.3)	3805 (69.5)	<.001
Yes	1595 (24.7)	1648 (30.5)	
Statin use^e^			
No	5244 (81.5)	4239 (77.7)	<.001
Yes	1190 (18.5)	1214 (22.3)	

Abbreviations: CRC, colorectal cancer; MET, metabolic equivalent of task; NA, not applicable; NSAIDs, nonsteroidal anti-inflammatory drugs.

^a^ Former defined as not smoking for at least 2 years prior to the interview.

^b^ Categories defined by quartiles among controls drinking alcohol.

^c^ Categories defined by quartiles of average lifetime MET-hours per week among controls.

^d^ NSAIDs use was defined as taking NSAIDs (including aspirin) at least 2 times per week for at least 1 year.

^e^ Defined as current regular use of statins 1 or more times per week for more than 1 year.

### Associations of BMI and Weight Change With CRC Risk According to the Time Point of Reported Weight

**Figure 2.**
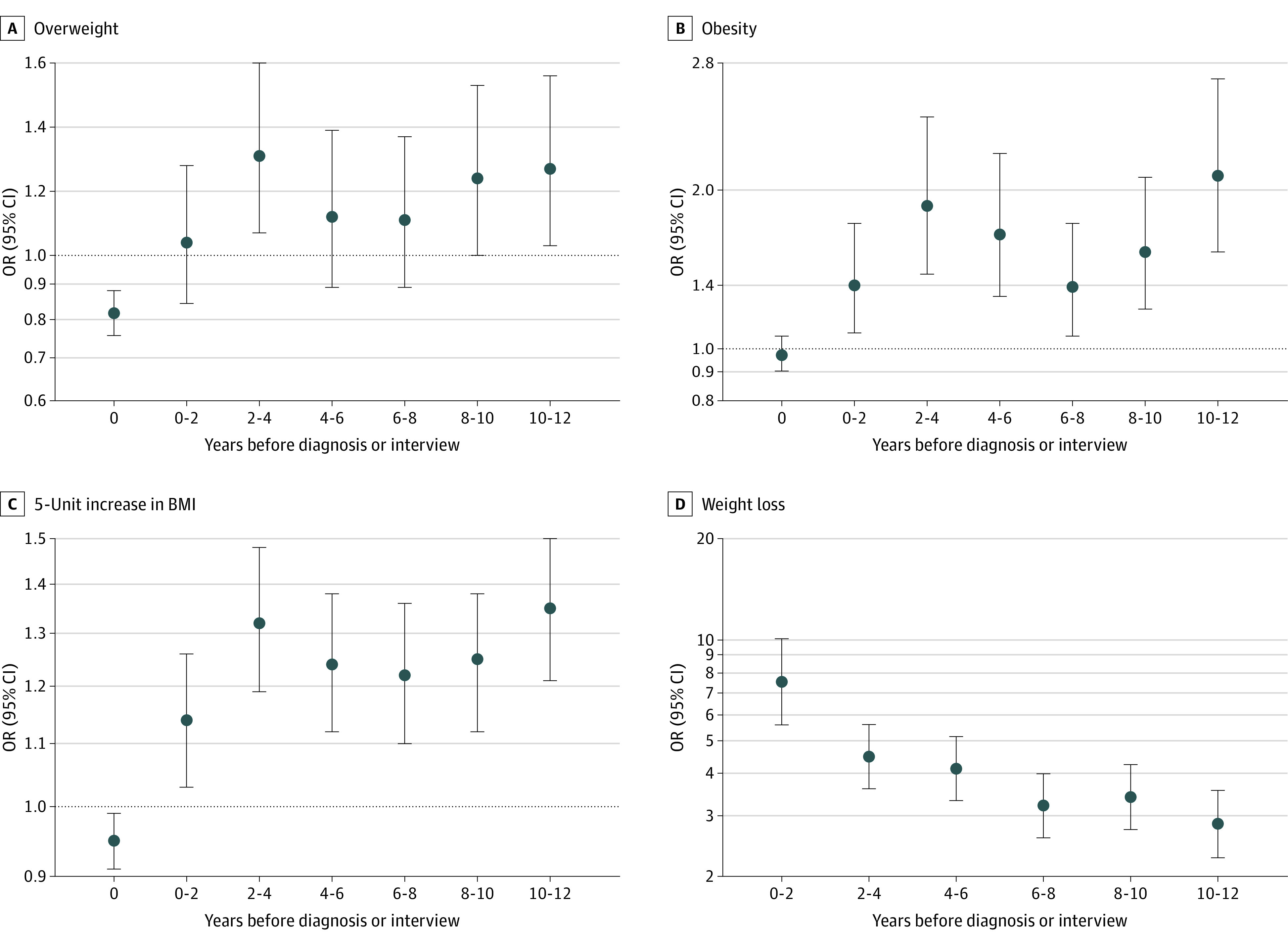
Association of Overweight, Obesity, and Weight Change With Colorectal Cancer Risk, Using Weights From Different Time Periods Before Diagnosis BMI indicates body mass index (calculated as weight in kilograms divided by height in meters squared); OR, odds ratio.

Table 2 and Figure 2 report the associations of overweight, obesity, a 5-unit increase in BMI, and weight change with CRC risk obtained by using either current weight or weight within different time windows before the time of diagnosis or interview. At the time of recruitment, 3998 cases (62.1%) and 3601 controls (66.0%) were overweight or obese (Table 2). No association (obesity) or even an inverse association (overweight, 5-unit increase) was observed between higher BMI and CRC risk when using current BMI. Conversely, increased risks were seen when using reported weight from several years ago. Estimates for overweight, obesity, and a 5-unit increase in BMI gradually increased when the analyses were based on weights from a longer time ago and reached maximum values of 1.31 (95% CI, 1.07-1.60), 2.09 (95% CI, 1.61-2.70), and 1.35 (95% CI, 1.21-1.50), respectively, when using weights from 8 to 10 or 10 to 12 years before diagnosis or interview. Weight loss (of 2 kg or more) from up to 2 years before diagnosis or interview was associated with a dramatic increase in CRC risk (OR, 7.52; 95% CI, 5.61-10.09), and the associations gradually decreased when using weights from earlier time frames. Weight gain was positively associated with CRC risk only when calculated using the reported weight for the period 0 to 2 years before diagnosis or interview. The ORs for the associations and trends for both BMI and weight change at different time frames before diagnosis or interview were similar across sex and CRC subsites (eTables 3, 4, 5, and 6 in Supplement 1). Estimates for weight change defined as 5% weight change followed the same pattern as when weight change was defined as a 2 kg or greater difference, but with higher ORs (5% weight loss from 0 to 2 years: OR, 11.14; 95% CI, 7.39-16.78; 5% weight gain from 0 to 2 years: OR, 2.00; 95% CI, 1.22-3.26) (eTable 7 in Supplement 1).

**Table 2.** Colorectal Cancer Risk According to Body Mass Index and Weight Change at Various Intervals Before Diagnosis or Interview

Time window	BMI category/weight change	Participants, No. (%)		OR (95% CI)	
		Cases	Controls	Model 1^a^	Model 2^b^
Diagnosis /interview	<18.5	127 (2.0)	29 (0.5)	3.47 (2.34-5.31)	3.25 (2.14-5.09)
	18.5 to <25	2309 (35.9)	1823 (33.4)	1 [Reference]	1 [Reference]
	25 to <30	2670 (41.5)	2526 (46.3)	0.83 (0.76-0.90)	0.82 (0.75-0.89)
	≥30	1328 (20.6)	1075 (19.7)	0.98 (0.88-1.08)	0.96 (0.86-1.08)
	Per 5-unit increase	NA	NA	0.94 (0.91-0.98)	0.95 (0.91-0.99)
0-2 y ago	<18.5	15 (1.2)	7 (0.6)	2.32 (0.93-5.76)	2.26 (0.85-5.95)
	18.5 to <25	395 (30.3)	409 (33.4)	1 [Reference]	1 [Reference]
	25 to <30	556 (44.2)	562 (46.7)	1.04)0.87-1.25)	1.04 (0.85-1.28)
	≥30	305 (24.3)	232 (19.3)	1.38 (1.11-1.73)	1.40 (1.10-1.79)
	Per 5-unit increase	NA	NA	1.12 (1.02-1.22)	1.14 (1.03-1.26)
	No change^c^	772 (42.6)	1040 (86.5)	1 [Reference]	1 [Reference]
	Loss ≥2 kg	389 (30.5)	78 (6.5)	6.87 (5.26-8.98)	7.52 (5.61-10.09)
	Gain ≥2 kg	96 (7.6)	85 (7.1)	1.55 (1.14-2.10)	1.54 (1.11-2.16)
2-4 y ago	<18.5	11 (0.9)	3 (0.3)	3.52 (0.97-12.78)	3.51 (0.90-13.71)
	18.5 to <25	337 (26.2)	330 (31.7)	1 [Reference]	1 [Reference]
	25 to <30	577 (44.9)	498 (47.8)	1.15 (0.94-1.40)	1.12 (0.90-1.39)
	≥30	361 (28.1)	210 (20.2)	1.72 (1.37-2.17)	1.72 (1.33-2.23)
	Per 5-unit increase	NA	NA	1.23 (1.13-1.35)	1.24 (1.12-1.38)
	No change^c^	483 (37.6)	661 (63.5)	1 [Reference]	1 [Reference]
	Loss ≥2 kg	652 (50.7)	186 (17.9)	4.83 (3.94-5.92)	4.52 (3.63-5.62)
	Gain ≥2 kg	151 (11.7)	194 (18.6)	1.06 (0.83-1.36)	1.08 (0.82-1.41)
4-6 y ago	<18.5	13 (1.0)	8 (0.8)	1.38 (0.56-3.37)	1.51 (0.56-4.07)
	18.5 to <25	762 (29.3)	349 (33.7)	1 [Reference]	1 [Reference]
	25 to <30	1182 (44.5)	483 (46.6)	1.10 (0.91-1.33)	1.11 (0.90-1.37)
	≥30	709 (25.3)	196 (18.9)	1.54 (1.23-1.94)	1.39 (1.08-1.79)
	Per 5-unit increase	NA	NA	1.25 (1.14-1.38)	1.22 (1.10-1.36)
	No change^c^	415 (32.1)	574 (51.2)	1 [Reference]	1 [Reference]
	Loss ≥2 kg	650 (50.3)	243 (21.7)	4.11 (3.37-5.01)	4.16 (3.35-5.18)
	Gain ≥2 kg	227 (17.6)	304 (27.1)	1.08 (0.86-1.34)	1.02 (0.80-1.29)
6-8 y ago	<18.5	10 (0.8)	7 (0.6)	1.51 (0.57-4.02)	1.63 (0.56-4.76)
	18.5 to <25	340 (27.4)	370 (33.3)	1 [Reference]	1 [Reference]
	25 to <30	571 (46.0)	516 (46.5)	1.21 (0.99-1.47)	1.24 (1.00-1.53)
	≥30	320 (25.8)	217 (19.5)	1.61 (1.28-2.02)	1.61 (1.25-2.08)
	Per 5-unit increase	NA	NA	1.23 (1.12-1.35)	1.25 (1.12-1.38)
	No change^c^	403 (32.5)	544 (49.0)	1 [Reference]	1 [Reference]
	Loss ≥2 kg	590 (47.5)	237 (21.4)	3.37 (2.77-4.12)	3.24 (2.60-4.02)
	Gain ≥2 kg	248 (20.0)	329 (29.6)	1.03 (0.83-1.27)	0.95 (0.75-1.20)
8-10 y ago	<18.5	5 (0.4)	6 (0.6)	0.90 (0.53-1.55)	0.69 (0.20-2.46)
	18.5 to <25	370 (28.8)	401 (37.7)	1 [Reference]	1 [Reference]
	25 to <30	581 (45.3)	479 (45.1)	1.32 (1.09-1.60)	1.27 (1.03-1.56)
	≥30	327 (25.5)	177 (16.7)	2.01 (1.59-2.54)	2.09 (1.61-2.70)
	Per 5-unit increase	NA	NA	1.32 (1.20-1.46)	1.35 (1.21-1.50)
	No change^c^	397 (31.7)	523 (47.6)	1 [Reference]	1 [Reference]
	Loss ≥2 kg	580 (46.3)	236 (21.5)	3.65 (2.97-4.49)	3.43 (2.75-4.28)
	Gain ≥2 kg	277 (22.1)	340 (30.9)	0.99 (0.81-1.22)	1.01 (0.81-1.27)
10-12 y ago	<18.5	11 (0.9)	7 (0.6)	1.89 (0.72-4.95)	1.87 (0.68-5.14)
	18.5 to <25	385 (30.6)	476 (39.6)	1 [Reference]	1 [Reference]
	25 to <30	582 (46.3)	542 (45.1)	1.35 (1.12-1.62)	1.31 (1.07-1.60)
	≥30	279 (22.2)	178 (14.8)	1.96 (1.55-2.48)	1.90 (1.47-2.46)
	Per 5-unit increase	NA	NA	1.32 (1.19-1.46)	1.32 (1.19-1.48)
	No change^c^	321 (25.5)	404 (33.6)	1 [Reference]	1 [Reference]
	Loss ≥2 kg	562 (44.7)	243 (20.2)	2.93 (2.37-3.61)	2.86 (2.27-3.59)
	Gain ≥2 kg	556 (29.8)	556 (46.2)	0.82 (0.67-1.00)	0.82 (0.66-1.02)

Abbreviations: BMI, body mass index (calculated as weight in kilograms divided by height in meters squared); NA, not applicable; OR, odds ratio.

^a^ Adjusted for age and sex.

^b^ Adjusted for age, sex, previous lower gastrointestinal endoscopy, colorectal cancer family history, education, smoking, alcohol consumption, nonsteroidal anti-inflammatory drugs use, physical activity, and statin use.

^c^ Within 2 kg.

Participants diagnosed with CRC experienced considerable prediagnostic weight loss. The odds of experiencing weight loss of 2 kg or more within the 2-year period prior to diagnosis or interview were estimated to be 7.52-fold (95% CI, 5.61-10.09) higher among cases than controls (11.14-fold increase [95% CI, 7.39-16.78] for 5% or more weight loss). Because prediagnostic weight loss is more pronounced closer to cancer diagnosis, ORs for weight loss since earlier time periods gradually decreased to 2.86 (95% CI, 2.27-3.59) for weight loss since 10 to 12 years before diagnosis or interview.

## Discussion

The role of overweight and obesity as risk factors for CRC has long been established.^1^ In this study, we provided evidence from a large population-based case-control study that lack of due consideration of prediagnostic weight loss can lead to a strong underestimation of the association of excess weight with CRC risk.

The mean sojourn time for CRC in the preclinical state has been consistently estimated to be about 3 to 6 years.^8,9,10^ Many patients with CRC experience weight loss during this period.^18^ However, most epidemiological studies investigating the association of excess weight with CRC risk ascertained body weight at a single point in time. In case-control studies, body weight is usually assessed shortly before or after cancer diagnosis, at which point the patients might have already lost considerable weight. In such studies, prediagnostic weight loss may have led to underestimation, absence, or even reversal of direction of the BMI-CRC association, as observed, for example, in 2 studies from 2020 on early onset CRC.^19,20^ In cohort studies, in which BMI is often objectively determined at baseline, underestimation of the BMI-CRC association may likewise be of concern because cancers diagnosed during the early years may have been present in the preclinical state and may have led to weight loss already at enrollment.

In our analysis of the DACHS study, a large population-based case-control study from Germany, the majority of patients reported a lower current weight than weight at their preceding decennial age, whereas an opposite pattern with a recent weight increase was seen among the majority of controls. These patterns point to considerable prediagnostic weight loss. Although the fact that the time interval between reported current weight and weight at the preceding decennial age varied between 1 and 10 years, limiting the comparability of weight loss across patients, it also offered unique opportunities to investigate weight loss during various time windows before diagnosis, and to examine how the timing of weight ascertainment may factor into observed effect estimates of overweight and obesity. Using a logistic regression model adjusted for potential confounders, the odds of experiencing weight loss of 2 kg or more within the 2-year period prior to diagnosis or interview were estimated to be 7.52-fold higher among cases than controls. Unsurprisingly, because prediagnostic weight loss is more pronounced closer to cancer diagnosis, ORs for weight loss since earlier time periods gradually decreased.

Our analysis demonstrates how strongly prediagnostic weight loss can affect estimates of the excess weight–CRC association. Had we looked only at the current BMI, the association of obesity with CRC risk would have been missed entirely. Moreover, for overweight and a 5-unit increase in BMI, we could have even come to the wrong conclusion that it has a protective effect on CRC risk. Looking at different data subsets based on available BMI data before diagnosis or interview, already moving from current BMI to the period of 0 to 2 years before diagnosis or interview led to a completely different pattern, with significant risk increases associated with obesity and a 5-unit increase in BMI. However, the estimated increase in risk for those with overweight remained very modest and statistically nonsignificant. As we gradually reduced the potential bias due to prediagnostic weight loss by shifting the window of reported former BMI further back in time, we saw an increase in the BMI-CRC association for all 3 measures of excess weight. The associations were especially pronounced when reported former BMI referred to a period of 8 to 10 or 10 to 12 years before diagnosis or interview.

To our knowledge, our study is among the first to specifically assess how the association of excess weight with CRC risk changes depending on the timing of the ascertainment of body weight. Previous case-control studies that looked at the current BMI or BMI relatively shortly before diagnosis may have severely underestimated the true magnitude of the association. Even though most more recent systematic reviews and meta-analyses only considered studies with a prospective cohort study design,^5,7,21,22^ limited follow-up times (implying that most cases occurred relatively shortly after weight ascertainment) and not excluding the first few years of follow-up would likely also have led to a considerable underestimation. Recently, in an umbrella review of meta-analyses and systematic reviews, we showed that none of the 18 reviews evaluating the BMI-CRC association considered prediagnostic weight loss in their analyses. According to the most recent systematic review and meta-analysis in 2021,^7^ the summary relative risks for obesity and 5-unit increase in BMI (based on 20 and 14 prospective cohort studies, respectively) were 1.31 (95% CI, 1.21-1.42) and 1.14 (95% CI, 1.09-1.20), respectively. These values are close to the estimates we obtained when looking at weight 0 to 2 years before diagnosis or interview in our case-control study, but considerably lower than the estimates obtained when looking at weight a longer time ago. However, a closer look at the individual primary studies included in this meta-analysis revealed that the majority of those studies (15 of 21) did not exclude any initial years of follow-up, and 9 out of 20 studies reporting on obesity had relatively short follow-up times (less than 10 years).^13^ Those that did exclude some first years of follow-up in their sensitivity analyses, did so mostly only for the first 1 or 2 years, and have rarely explicitly reported results from such analyses. Our findings suggest that more rigorous approaches may be needed to fully disclose the association of excess weight with CRC risk, which may be substantially larger than previously assumed. In particular, time periods should be excluded from risk estimates in which weight loss is a marker for strongly increased CRC risk.

### Strengths and Limitations

Strengths of this study include its population-based study design, large sample size, detailed ascertainment of body weight at various ages, and its comprehensive adjustment for potential confounders. Nonetheless, our analysis had several limitations. First, weight was self-reported, which may go along with imperfect recall, particularly with respect to weight at earlier ages. Such imperfect recall would most likely have affected both cases and controls in a similar manner, and thereby most likely have led to the underestimation of BMI-CRC association. On the other hand, healthier and better-educated people may have been more likely to have participated as controls, which means that people with overweight and obesity might have been underrepresented among the controls. This selection bias would have affected the results toward overestimation. A further limitation of the study is the exclusive focus on adult BMI, while overweight, obesity, and weight gain in early life might also be a particularly informative risk predictor for CRC.^23,24,25,26^ Future studies should also consider alternative indicators of adiposity, such as waist circumference and waist-to-hip ratio.

## Conclusion

In summary, findings of this large-scale population-based case-control study illustrate the dramatic change of BMI as a risk factor associated with CRC, depending on whether the period of potential prediagnostic weight loss is accounted for or not. Further studies examining the association between excess body weight and CRC risk should put more effort into avoiding bias due to cancer-related weight loss before diagnosis. While we demonstrated that prediagnostic weight loss is a major concern for CRC, it appears plausible to assume that it may play a similarly important role for other cancers and noncancer diseases associated with overweight and obesity, which should be addressed in further research. Our study also points to involuntary prediagnostic weight loss as a potential marker for early detection of CRC. Most importantly, however, our results emphasize the importance of interventions aimed at preventing and managing overweight and obesity, which are steadily rising in prevalence in many parts of the world, and which may factor more substantially into CRC risk and other obesity-related diseases than suggested by existing epidemiological evidence.

## Notes

Supplement 1. **eFigure.** Flow Chart Showing the Selection of Study Population **eTable 1.** Available Information on Weight at Various Time Points According to Age at Diagnosis of Cases/Age at Interview of Controls **eTable 2.** Time Windows Before Diagnosis Used for Analysis, Using Available Information on Weight at Various Time Points According to Age at Diagnosis of Cases/Age at the Interview of Controls **eTable 3.** Men—CRC Risk According to BMI and Weight Change at Various Intervals Before Diagnosis/Interview **eTable 4.** Women—CRC Risk According to BMI and Weight Change at Various Intervals Before Diagnosis/Interview **eTable 5.** Colon—Risk According to BMI and Weight Change at Various Intervals Before Diagnosis/Interview **eTable 6.** Rectum—Risk According to BMI and Weight Change at Various Intervals Before Diagnosis/Interview **eTable 7.** CRC Risk According to ≥5% Weight Change Since Different Intervals Before Diagnosis/Interview

Supplement 2.
Data Sharing Statement

## References

[1] Renehan AG, Tyson M, Egger M, Heller RF, Zwahlen M. Body-mass index and incidence of cancer: a systematic review and meta-analysis of prospective observational studies. Lancet. 2008;371(9612):569-578. doi:10.1016/S0140-6736(08)60269-X 18280327

[2] World Health Organization. Obesity: preventing and managing the global epidemic. Published 2000. Accessed March 31, 2022. https://apps.who.int/iris/handle/10665/42330 11234459

[3] Abarca-Gómez L, Abdeen ZA, Hamid ZA, ; NCD Risk Factor Collaboration (NCD-RisC). Worldwide trends in body-mass index, underweight, overweight, and obesity from 1975 to 2016: a pooled analysis of 2416 population-based measurement studies in 128·9 million children, adolescents, and adults. Lancet. 2017;390(10113):2627-2642. doi:10.1016/S0140-6736(17)32129-3 29029897 PMC5735219

[4] World Health Organization. Obesity and overweight. Accessed April 21, 2022. https://www.who.int/news-room/fact-sheets/detail/obesity-and-overweight

[5] Garcia H, Song M. Early-life obesity and adulthood colorectal cancer risk: a meta-analysis. Rev Panam Salud Publica. 2019;43:e3. doi:10.26633/RPSP.2019.3 31093227 PMC6393738

[6] Lei X, Song S, Li X, Geng C, Wang C. Excessive body fat at a young age increases the risk of colorectal cancer: a systematic review and meta-analysis. Nutr Cancer. 2021;73(9):1601-1612. doi:10.1080/01635581.2020.1804951 32791859

[7] Zhang C, Cheng Y, Luo D, . Association between cardiovascular risk factors and colorectal cancer: a systematic review and meta-analysis of prospective cohort studies. EClinicalMedicine. 2021;34:100794. doi:10.1016/j.eclinm.2021.100794 33997727 PMC8102710

[8] Launoy G, Smith TC, Duffy SW, Bouvier V. Colorectal cancer mass-screening: estimation of faecal occult blood test sensitivity, taking into account cancer mean sojourn time. Int J Cancer. 1997;73(2):220-224. doi:10.1002/(SICI)1097-0215(19971009)73:2<220::AID-IJC10>3.0.CO;2-J 9335446

[9] Prevost TC, Launoy G, Duffy SW, Chen HH. Estimating sensitivity and sojourn time in screening for colorectal cancer: a comparison of statistical approaches. Am J Epidemiol. 1998;148(6):609-619. doi:10.1093/oxfordjournals.aje.a009687 9753016

[10] Brenner H, Altenhofen L, Katalinic A, Lansdorp-Vogelaar I, Hoffmeister M. Sojourn time of preclinical colorectal cancer by sex and age: estimates from the German national screening colonoscopy database. Am J Epidemiol. 2011;174(10):1140-1146. doi:10.1093/aje/kwr188 21984657

[11] Bapuji SB, Sawatzky JAV. Understanding weight loss in patients with colorectal cancer: a human response to illness. Oncol Nurs Forum. 2010;37(3):303-310. doi:10.1188/10.ONF.303-310 20439214

[12] van Zutphen M, Geelen A, Boshuizen HC, . Pre-to-post diagnosis weight trajectories in colorectal cancer patients with non-metastatic disease. Support Care Cancer. 2019;27(4):1541-1549. doi:10.1007/s00520-018-4560-z 30484014 PMC6394719

[13] Mandic M, Li H, Safizadeh F, Niedermaier T, Hoffmeister M, Brenner H. Is the association of overweight and obesity with colorectal cancer underestimated? an umbrella review of systematic reviews and meta-analyses. Eur J Epidemiol. 2023;38(2):135-144. doi:10.1007/s10654-022-00954-6 36680645 PMC9905196

[14] Brenner H, Chang-Claude J, Seiler CM, Rickert A, Hoffmeister M. Protection from colorectal cancer after colonoscopy: a population-based, case-control study. Ann Intern Med. 2011;154(1):22-30. doi:10.7326/0003-4819-154-1-201101040-00004 21200035

[15] Brenner H, Chang-Claude J, Rickert A, Seiler CM, Hoffmeister M. Risk of colorectal cancer after detection and removal of adenomas at colonoscopy: population-based case-control study. J Clin Oncol. 2012;30(24):2969-2976. doi:10.1200/JCO.2011.41.3377 22826281

[16] Hoffmeister M, Bläker H, Kloor M, . Body mass index and microsatellite instability in colorectal cancer: a population-based study. Cancer Epidemiol Biomarkers Prev. 2013;22(12):2303-2311. doi:10.1158/1055-9965.EPI-13-0239 24127414

[17] van Buuren S, Groothuis-Oudshoorn K. mice: multivariate imputation by chained equations in R. J Stat Soft. 2011;45:1-67. doi:10.18637/jss.v045.i03

[18] Hamilton W, Round A, Sharp D, Peters TJ. Clinical features of colorectal cancer before diagnosis: a population-based case-control study. Br J Cancer. 2005;93(4):399-405. doi:10.1038/sj.bjc.6602714 16106247 PMC2361578

[19] Low EE, Demb J, Liu L, . Risk factors for early-onset colorectal cancer. Gastroenterology. 2020;159(2):492-501.e7. doi:10.1053/j.gastro.2020.01.004 31926997 PMC7343609

[20] Gausman V, Dornblaser D, Anand S, . Risk factors associated with early-onset colorectal cancer. Clin Gastroenterol Hepatol. 2020;18(12):2752-2759.e2. doi:10.1016/j.cgh.2019.10.009 31622737 PMC7153971

[21] Fang X, Wei J, He X, . Quantitative association between body mass index and the risk of cancer: a global Meta-analysis of prospective cohort studies. Int J Cancer. 2018;143(7):1595-1603. doi:10.1002/ijc.31553 29696630

[22] Abar L, Vieira AR, Aune D, . Height and body fatness and colorectal cancer risk: an update of the WCRF-AICR systematic review of published prospective studies. Eur J Nutr. 2018;57(5):1701-1720. doi:10.1007/s00394-017-1557-1 29080978 PMC6060816

[23] Aleksandrova K, Pischon T, Buijsse B, . Adult weight change and risk of colorectal cancer in the European Prospective Investigation into Cancer and Nutrition. Eur J Cancer. 2013;49(16):3526-3536. doi:10.1016/j.ejca.2013.06.021 23867126

[24] Renehan AG, Flood A, Adams KF, . Body mass index at different adult ages, weight change, and colorectal cancer risk in the National Institutes of Health-AARP Cohort. Am J Epidemiol. 2012;176(12):1130-1140. doi:10.1093/aje/kws192 23186750 PMC6287246

[25] Song M, Hu FB, Spiegelman D, . Long-term status and change of body fat distribution, and risk of colorectal cancer: a prospective cohort study. Int J Epidemiol. 2016;45(3):871-883. doi:10.1093/ije/dyv177 26403814 PMC5005941

[26] Li H, Boakye D, Chen X, . Associations of body mass index at different ages with early-onset colorectal cancer. Gastroenterology. 2022;162(4):1088-1097.e3. doi:10.1053/j.gastro.2021.12.239 34914944

