# Supplementary material for: Association of Overweight, Obesity, and Recent Weight Loss With Colorectal Cancer Risk
Source: JAMA Netw Open. 2023 Apr 21;6(4):e239556. doi: 10.1001/jamanetworkopen.2023.9556 (PMC10122181; doi:10.1001/jamanetworkopen.2023.9556)
Supplement: Supplement 1. — eFigure. Flow Chart Showing the Selection of Study Population eTable 1. Available Information on Weight at Various Time Points According to Age at Diagnosis of Cases/Age at Interview of Controls eTable 2. Time Windows Before Diagnosis Used for Analysis, Using Available Information on Weight at Various Time Points According to Age at Diagnosis of Cases/Age at the Interview of Controls eTable 3. Men—CRC Risk According to BMI and Weight Change at Various Intervals Before Diagnosis/Interview eTable 4. Women—CRC Risk According to BMI and Weight Change at Various Intervals Before Diagnosis/Interview eTable 5. Colon—Risk According to BMI and Weight Change at Various Intervals Before Diagnosis/Interview eTable 6. Rectum—Risk According to BMI and Weight Change at Various Intervals Before Diagnosis/Interview eTable 7. CRC Risk According to ≥5% Weight Change Since Different Intervals Before Diagnosis/Interview [file jamanetwopen-e239556-s001.pdf]

Supplemental Online Content

Mandic M, Safizadeh F, Niedermaier T, Hoffmeister M, Brenner H. Association of overweight, obesity and recent weight loss with colorectal cancer risk. *JAMA Netw Open.* 2023;6(4):e239556. doi:10.1001/jamanetworkopen.2023.9556

eFigure. Flow Chart Showing the Selection of Study Population

eTable 1. Available Information on Weight at Various Time Points According to Age at Diagnosis of Cases/Age at Interview of Controls

eTable 2. Time Windows Before Diagnosis Used for Analysis, Using Available Information on Weight at Various Time Points According to Age at Diagnosis of Cases/Age at the Interview of Controls

eTable 3. Men—CRC Risk According to BMI and Weight Change at Various Intervals Before Diagnosis/Interview

eTable 4. Women—CRC Risk According to BMI and Weight Change at Various Intervals Before Diagnosis/Interview

eTable 5. Colon—Risk According to BMI and Weight Change at Various Intervals Before Diagnosis/Interview

eTable 6. Rectum—Risk According to BMI and Weight Change at Various Intervals Before Diagnosis/Interview

eTable 7. CRC Risk According to ≥5% Weight Change Since Different Intervals Before Diagnosis/Interview

This supplemental material has been provided by the authors to give readers additional information about their work.

© 2023 Mandic M et al. *JAMA Network Open.*

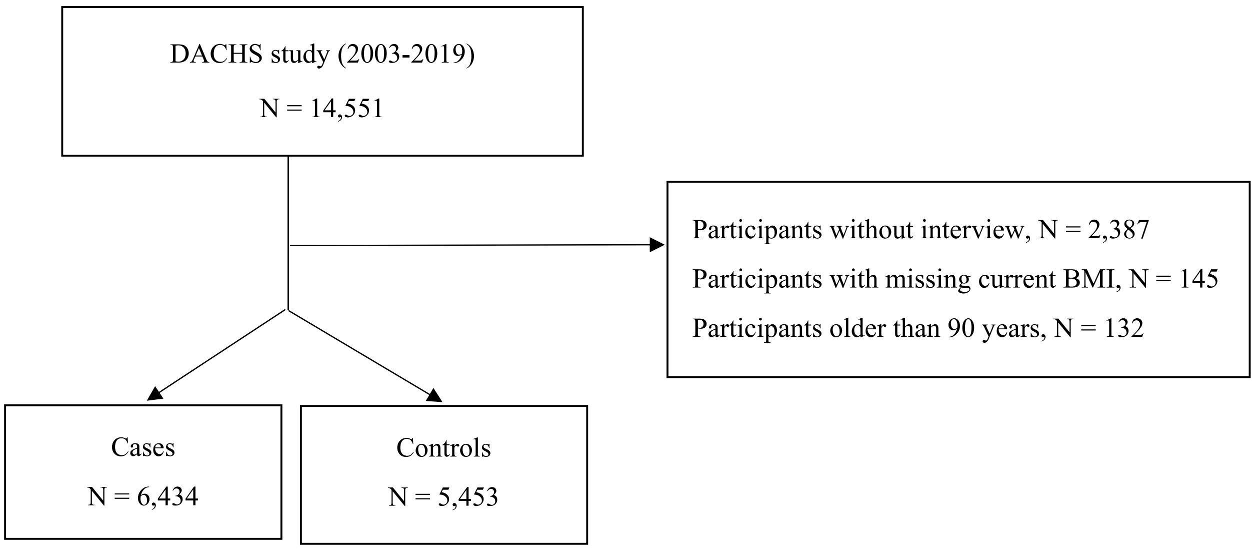

eFigure. Flow chart showing the selection of study population*.

* Data were imputed for the following missing variables:
Education: data missing for 12 cases and 8 controls.
Smoking status: data missing for 45 cases and 19 controls.
Alcohol consumption: data missing for 34 cases and 31 controls.
Physical activity: data missing for 86 cases and 11 controls.
History of CRC in 1st-degree relative: data missing for 7 cases and 3 controls.
Previous lower gastrointestinal endoscopy: data missing for 2 cases.
Diabetes: data missing for 38 cases and 26 controls.
Statin use: data missing for 3 cases and 5 cases.

© 2023 Mandic M et al. JAMA Network Open.

eTable 1. Available information on weight at various time points according to age at diagnosis of cases/age at interview of controls.

**Ages at diagnosis or interview**	**Available weight information**												
	current	0-1 y ago	1-2 y ago	2-3 y ago	3-4 y ago	4-5 y ago	5-6 y ago	6-7 y ago	7-8 y ago	8-9 y ago	9-10 y ago	10-11 y ago	11-12 y ago
30, 40, 50, 60, 70, 80	X	X										X	
31, 41, 51, 61, 71, 81	X		X										X
32, 42, 52, 62, 72, 82	X			X									
33, 43, 53, 63, 73, 83	X				X								
34, 44, 54, 64, 74, 84	X					X							
35, 45, 55, 65, 75, 85	X						X						
36, 46, 56, 66, 76, 86	X							X					
37, 47, 57, 67, 77, 87	X								X				
38, 48, 58, 68, 78, 88	X									X			
39, 49, 59, 69, 79, 89	X										X		

Abbreviations: y = year(s).

eTable 2. Time windows before diagnosis used for analysis, using available information on weight at various time points according to age at diagnosis of cases/age at the interview of controls.

	**Available weight information**						
**Ages at diagnosis or interview**	current	0-2 years ago	2-4 years ago	4-6 years ago	6-8 years ago	8-10 years ago	10-12 years ago
30, 40, 50, 60, 70, 80	X	X					X
31, 41, 51, 61, 71, 81	X	X					X
32, 42, 52, 62, 72, 82	X		X				
33, 43, 53, 63, 73, 83	X		X				
34, 44, 54, 64, 74, 84	X			X			
35, 45, 55, 65, 75, 85	X			X			
36, 46, 56, 66, 76, 86	X				X		
37, 47, 57, 67, 77, 87	X				X		
38, 48, 58, 68, 78, 88	X					X	
39, 49, 59, 69, 79, 89	X					X	

© 2023 Mandic M et al. JAMA Network Open.

eTable 3. Men - CRC risk according to BMI and weight change at various intervals before diagnosis/interview

Time window	BMI category / weight change	Cases No (%)	Controls No (%)	OR (95% CI)	
				Model 1[b]	Model 2[c]
**Diagnosis /interview**	<18.5	29 (0.8)	4 (0.1)	5.37 [2.11-18.16]	6.04 [2.22-21.24]
	18.5 to <25	2309 (32.1)	919 (27.7)	[Reference]	[Reference]
	25 to <30	2670 (46.1)	1720 (51.9)	0.77 [0.69-0.85]	0.76 [0.67-0.85]
	≥30	1328 (21.0)	673 (20.3)	0.90 [0.79-1.03]	0.87 [0.75-1.01]
	Per 5-unit increase			0.90 [0.85-0.96]	0.91 [0.85-0.97]
**0-2 years ago**	<18.5	3 (0.4)	1 (0.1)		
	18.5 to <25	195 (25.8)	198 (27.3)	[Reference]	[Reference]
	25 to <30	365 (48.3)	386 (53.3)	0.95 [0.74-1.22]	0.97 [0.74-1.27]
	≥30	193 (25.5)	139 (19.2)	1.41 [1.05-1.89]	1.43 [1.03-1.99]
	Per 5-unit increase			1.09 [0.97-1.07]	1.13 [0.98-1.30]
	No change[a]	437 (57.8)	621 (85.8)	[Reference]	[Reference]
	Loss ≥2 kg	260 (34.4)	54 (7.5)	6.84 [4.96-9.45]	7.49 [5.26-10.68]
	Gain ≥2 kg	59 (7.8)	49 (6.8)	1.75 [1.16-2.64]	1.57 [1.00-2.47]
**2-4 years ago**	<18.5	3 (0.4)	1 (0.2)		
	18.5 to <25	150 (19.9)	150 (23.7)	[Reference]	[Reference]
	25 to <30	386 (51.3)	341 (53.9)	1.11 [0.85-1.46]	1.03 [0.76-1.40]
	≥30	213 (28.3)	141 (22.3)	1.51 [1.10-2.06]	1.46 [1.03-2.07]
	Per 5-unit increase			1.21 [1.07-1.38]	1.19 [1.03-1.38]
	No change[a]	271 (36.0)	401 (63.3)	[Reference]	[Reference]
	Loss ≥2 kg	396 (52.7)	123 (19.4)	4.85 [3.75-6.26]	4.47 [3.37-5.92]
	Gain ≥2 kg	85 (11.3)	109 (17.2)	1.19 [0.86-1.65]	1.19 [0.83-1.71]
**4-6 years ago**	<18.5	4 (0.5)	2 (0.3)		
	18.5 to <25	178 (22.0)	164 (26.5)	[Reference]	[Reference]
	25 to <30	403 (49.9)	328 (53.0)	1.13 [0.88-1.47]	1.21 [0.90-1.62]
	≥30	223 (27.6)	125 (20.2)	1.64 [1.21-2.23]	1.51 [1.07-2.14]
	Per 5-unit increase			1.30 [1.14-1.48]	1.28 [1.11-1.49]
	No change[a]	260 (32.2)	330 (53.3)	[Reference]	[Reference]
	Loss ≥2 kg	420 (52.0)	143 (23.1)	3.75 [2.92-4.82]	3.82 [2.89-5.06]
	Gain ≥2 kg	128 (15.8)	146 (23.6)	1.12 [0.84-1.50]	1.05 [0.76-1.46]
**6-8 years ago**	<18.5	0 (0.0)	2 (0.3)		
	18.5 to <25	173 (22.6)	187 (26.6)	[Reference]	[Reference]
	25 to <30	390 (50.8)	374 (53.3)	1.12 [0.87-1.44]	1.13 [0.86-1.50]
	≥30	204 (26.6)	139 (19.8)	1.58 [1.17-2.13]	1.53 [1.09-2.14]
	Per 5-unit increase			1.30 [1.14-1.49]	1.31 [1.13-1.52]
	No change[a]	235 (30.6)	343 (48.9)	[Reference]	[Reference]
	Loss ≥2 kg	386 (50.3)	149 (21.2)	3.77 [2.93-4.85]	3.66 [2.77-4.83]
	Gain ≥2 kg	146 (19.0)	210 (29.9)	1.01 [0.77-1.33]	0.96 [0.71-1.30]
**8-10 years ago**	<18.5	0 (0.0)	0 (0.0)		
	18.5 to <25	170 (22.0)	207 (32.4)	[Reference]	[Reference]
	25 to <30	400 (51.7)	321 (50.3)	1.53 [1.19-1.97]	1.45 [1.09-1.92]
	≥30	204 (26.4)	110 (17.2)	2.28 [1.67-3.10]	2.37 [1.68-3.36]
	Per 5-unit increase			1.42 [1.23-1.64]	1.44 [1.23-1.69]
	No change[a]	219 (28.3)	294 (46.1)	[Reference]	[Reference]
	Loss ≥2 kg	377 (48.7)	125 (19.6)	4.08 [3.12-5.34]	3.84 [2.87-5.14]
	Gain ≥2 kg	178 (23.0)	219 (34.3)	1.04 [0.80-1.36]	0.98 [0.73-1.32]
**10-12 years ago**	<18.5	1 (0.1)	1 (0.1)		
	18.5 to <25	184 (24.3)	231 (31.9)	[Reference]	[Reference]
	25 to <30	395 (52.2)	382 (52.8)	1.30 [1.02-1.65]	1.26 [0.97-1.64]
	≥30	176 (23.3)	110 (15.2)	2.00 [1.47-2.73]	1.96 [1.39-2.75]
	Per 5-unit increase			1.37 [1.19-1.58]	1.37 [1.17-1.61]
	No change[a]	183 (24.2)	253 (34.9)	[Reference]	[Reference]
	Loss ≥2 kg	362 (47.9)	149 (20.6)	3.37 [2.57-4.41]	3.47 [2.58-4.67]
	Gain ≥2 kg	322 (27.9)	322 (44.5)	0.88 [0.68-1.15]	0.95 [0.72-1.27]

[a] Within ± 2 kg.
[b] Adjusted for age.
[c] Adjusted for age, previous lower gastrointestinal endoscopy, CRC family history, education, smoking, alcohol consumption, NSAIDs use, physical activity, and statin use.
Abbreviations: BMI = body mass index, CRC = colorectal cancer, NSAIDs = nonsteroidal anti-inflammatory drugs, OR = odds ratio.

© 2023 Mandic M et al. JAMA Network Open.

eTable 4. Women - CRC risk according to BMI and weight change at various intervals before diagnosis/interview

Time window	BMI category / weight change	Cases No (%)	Controls No (%)	OR (95% CI)	
				Model 1[b]	Model 2[c]
Diagnosis /interview	<18.5	98 (3.8)	25 (1.2)	3.30 [2.14-5.27]	2.97 [1.88-4.87]
	18.5 to <25	1071 (41.6)	904 (42.3)	[Reference]	[Reference]
	25 to <30	891 (34.6)	806 (37.7)	0.92 [0.81-1.05]	0.89 [0.77-1.03]
	≥30	517 (20.1)	402 (18.8)	1.08 [0.92-1.27]	1.08 [0.91-1.28]
	Per 5-unit increase			0.97 [0.91-1.03]	0.97 [0.91-1.04]
0-2 years ago	<18.5	12 (2.4)	6 (1.3)		
	18.5 to <25	184 (36.7)	203 (42.4)	[Reference]	[Reference]
	25 to <30	193 (38.5)	176 (36.7)	1.20 [0.90-1.61]	1.16 [0.85-1.59]
	≥30	112 (22.4)	94 (19.6)	1.32 [0.94-1.85]	1.31 [0.90-1.92]
	Per 5-unit increase			1.15 [1.01-1.31]	1.14 [0.99-1.31]
	No change[a]	334 (66.7)	419 (87.5)	[Reference]	[Reference]
	Loss ≥2 kg	127 (25.3)	23 (4.8)	7.02 [4.37-11.28]	7.89 [4.72-13.20]
	Gain ≥2 kg	40 (8.0)	37 (7.7)	1.30 [0.81-2.10]	1.50 [0.89-2.51]
2-4 years ago	<18.5	8 (1.5)	2 (0.5)		
	18.5 to <25	187 (35.0)	181 (44.4)	[Reference]	[Reference]
	25 to <30	191 (35.8)	157 (38.5)	1.17 [0.87-1.58]	1.17 [0.84-1.63]
	≥30	148 (27.7)	68 (16.7)	2.10 [1.48-3.00]	2.19 [1.47-3.26]
	Per 5-unit increase			1.26 [1.10-1.43]	1.30 [1.12-1.51]
	No change[a]	215 (40.3)	260 (63.7)	[Reference]	[Reference]
	Loss ≥2 kg	254 (47.6)	63 (15.4)	4.86 [3.48-6.78]	4.73 [3.31-6.76]
	Gain ≥2 kg	65 (12.2)	85 (20.8)	0.91 [0.62-1.32]	0.94 [0.63-1.42]
4-6 years ago	<18.5	9 (0.5)	6 (1.4)		
	18.5 to <25	223 (39.9)	185 (44.4)	[Reference]	[Reference]
	25 to <30	204 (36.5)	155 (37.2)	1.07 [0.80-1.42]	0.99 [0.72-1.36]
	≥30	123 (22.0)	71 (17.0)	1.42 [1.00-2.02]	1.32 [0.89-1.94]
	Per 5-unit increase			1.20 [1.05-1.37]	1.17 [1.00-1.36]
	No change[a]	186 (33.3)	227 (54.4)	[Reference]	[Reference]
	Loss ≥2 kg	278 (49.7)	71 (17.0)	4.79 [3.46-6.64]	4.78 [3.36-6.81]
	Gain ≥2 kg	95 (17.0)	119 (28.5)	1.03 [0.73-1.45]	0.93 [0.64-1.35]
6-8 years ago	<18.5	10 (2.1)	5 (1.2)		
	18.5 to <25	167 (35.2)	183 (44.9)	[Reference]	[Reference]
	25 to <30	181 (38.2)	142 (34.8)	1.36 [1.00-1.85]	1.34 [0.95-1.88]
	≥30	116 (24.5)	78 (19.1)	1.60 [1.12-2.29]	1.66 [1.11-2.50]
	Per 5-unit increase			1.17 [1.02-1.33]	1.17 [1.01-1.36]
	No change[a]	168 (35.4)	202 (49.5)	[Reference]	[Reference]
	Loss ≥2 kg	204 (43.0)	88 (21.6)	2.78 [2.01-3.84]	2.66 [1.87-3.78]
	Gain ≥2 kg	102 (21.5)	118 (28.9)	1.06 [0.75-1.49]	0.93 [0.64-1.36]
8-10 years ago	<18.5	5 (1.0)	6 (1.4)		
	18.5 to <25	200 (39.3)	194 (45.6)	[Reference]	[Reference]
	25 to <30	181 (35.6)	158 (37.2)	1.07 [0.80-1.44]	1.07 [0.77-1.48]
	≥30	123 (24.2)	67 (15.8)	1.73 [1.21-2.48]	1.91 [1.28-2.85]
	Per 5-unit increase			1.23 [1.07-1.41]	1.28 [1.10-1.49]
	No change[a]	153 (30.1)	180 (42.4)	[Reference]	[Reference]
	Loss ≥2 kg	236 (46.4)	90 (21.2)	3.07 [2.22-4.25]	2.96 [2.09-4.21]
	Gain ≥2 kg	120 (23.6)	155 (36.5)	0.92 [0.67-1.28]	1.04 [0.73-1.48]
10-12 years ago	<18.5	10 (2.0)	6 (1.3)		
	18.5 to <25	201 (40.1)	245 (51.1)	[Reference]	[Reference]
	25 to <30	187 (37.3)	160 (33.4)	1.45 [1.09-1.94]	1.39 [1.01-1.90]
	≥30	103 (20.6)	68 (14.2)	1.88 [1.30-2.70]	1.77 [1.18-2.66]
	Per 5-unit increase			1.28 [1.11-1.47]	1.27 [1.09-1.49]
	No change[a]	138 (27.5)	151 (31.5)	[Reference]	[Reference]
	Loss ≥2 kg	200 (39.9)	94 (19.6)	2.33 [1.67-3.27]	2.14 [1.49-3.09]
	Gain ≥2 kg	163 (32.5)	234 (48.9)	0.73 [0.53-1.00]	0.67 [0.47-0.94]

[a] Within ± 2 kg.
[b] Adjusted for age.
[c] Adjusted for age, previous lower gastrointestinal endoscopy, CRC family history, education, smoking, alcohol consumption, NSAIDs use, physical activity, and statin use.
Abbreviations: BMI = body mass index, CRC = colorectal cancer, NSAIDs = nonsteroidal anti-inflammatory drugs, OR = odds ratio.

© 2023 Mandic M et al. JAMA Network Open.

eTable 5. Colon - risk according to BMI and weight change at various intervals before diagnosis/interview

Time window	BMI category / weight change	Cases No (%)	Controls No (%)	OR (95% CI)	
				Model 1[b]	Model 2[c]
**Diagnosis /interview**	<18.5	74 (1.9)	29 (0.5)	3.25 [2.12-5.11]	3.03 [1.93-4.87]
	18.5 to <25	1370 (34.8)	1823 (33.4)	[Reference]	[Reference]
	25 to <30	1616 (41.1)	2526 (46.3)	0.86 [0.78-0.94]	0.84 [0.76-0.93]
	≥30	872 (22.2)	1075 (19.7)	1.10 [0.98-1.24]	1.06 [0.94-1.20]
	Per 5-unit increase			0.98 [0.94-1.03]	0.98 [0.93-1.03]
**0-2 years ago**	<18.5	5 (0.7)	7 (0.6)		
	18.5 to <25	224 (29.5)	401 (33.3)	[Reference]	[Reference]
	25 to <30	339 (44.7)	562 (46.7)	1.08 [0.87-1.34]	1.07 [0.85-1.35]
	≥30	191 (25.2)	233 (19.4)	1.48 [1.15-1.91]	1.46 [1.11-1.92]
	Per 5-unit increase			1.15 [1.04-1.28]	1.15 [1.03-1.29]
	No change[a]	477 (62.8)	1045 (86.9)	[Reference]	[Reference]
	Loss ≥2 kg	224 (29.5)	76 (6.3)	6.64 [4.97-8.86]	6.90 [5.05-9.44]
	Gain ≥2 kg	58 (7.6)	82 (6.8)	1.52 [1.06-2.17]	1.55 [1.06-2.26]
**2-4 years ago**	<18.5	5 (0.6)	3 (0.3)		
	18.5 to <25	202 (25.9)	330 (31.7)	[Reference]	[Reference]
	25 to <30	335 (42.9)	499 (47.9)	1.14 [0.91-1.44]	1.09 [0.85-1.40]
	≥30	239 (30.6)	209 (20.1)	1.96 [1.51-2.55]	1.92 [1.44-2.55]
	Per 5-unit increase			1.32 [1.19-1.45]	1.31 [1.17-1.46]
	No change[a]	293 (37.5)	665 (63.9)	[Reference]	[Reference]
	Loss ≥2 kg	396 (50.7)	184 (17.7)	4.92 [3.93-6.17]	4.63 [3.64-5.89]
	Gain ≥2 kg	92 (11.8)	192 (18.4)	1.12 [0.83-1.50]	1.10 [0.80-1.50]
**4-6 years ago**	<18.5	10 (1.2)	8 (0.8)		
	18.5 to <25	243 (29.3)	349 (33.7)	[Reference]	[Reference]
	25 to <30	371 (44.8)	483 (46.6)	1.13 [0.91-1.40]	1.15 [0.91-1.46]
	≥30	205 (24.7)	196 (18.9)	1.53 [1.18-1.98]	1.41 [1.06-1.87]
	Per 5-unit increase			1.25 [1.12-1.39]	1.24 [1.10-1.39]
	No change[a]	266 (32.1)	556 (53.7)	[Reference]	[Reference]
	Loss ≥2 kg	414 (49.9)	215 (20.8)	4.12 [3.30-5.14]	4.34 [3.41-5.53]
	Gain ≥2 kg	149 (18.0)	265 (25.6)	1.26 [0.98-1.63]	1.17 [0.89-1.55]
**6-8 years ago**	<18.5	7 (0.9)	7 (0.6)		
	18.5 to <25	202 (26.5)	371 (33.4)	[Reference]	[Reference]
	25 to <30	343 (45.0)	515 (46.4)	1.21 [0.96-1.51]	1.25 [0.98-1.59]
	≥30	211 (27.7)	217 (19.5)	1.79 [1.38-2.32]	1.79 [1.34-2.37]
	Per 5-unit increase			1.26 [1.14-1.41]	1.27 [1.13-1.43]
	No change[a]	242 (31.7)	544 (49.0)	[Reference]	[Reference]
	Loss ≥2 kg	370 (48.5)	237 (21.4)	3.52 [2.82-4.41]	3.34 [2.63-4.26]
	Gain ≥2 kg	151 (19.8)	329 (29.6)	1.10 [0.86-1.41]	0.99 [0.76-1.30]
**8-10 years ago**	<18.5	4 (0.5)	6 (0.6)		
	18.5 to <25	229 (28.6)	401 (37.7)	[Reference]	[Reference]
	25 to <30	358 (44.8)	479 (45.1)	1.32 [1.07-1.65]	1.30 [1.03-1.64]
	≥30	209 (26.1)	177 (16.7)	2.09 [1.61-2.71]	2.15 [1.62-2.85]
	Per 5-unit increase			1.35 [1.21-1.51]	1.37 [1.22-1.54]
	No change[a]	230 (28.7)	474 (44.6)	[Reference]	[Reference]
	Loss ≥2 kg	368 (46.0)	215 (20.2)	3.50 [2.78-4.41]	3.46 [2.71-4.42]
	Gain ≥2 kg	202 (25.2)	374 (35.2)	1.13 [0.89-1.43]	1.17 [0.91-1.50]
**10-12 years ago**	<18.5	5 (0.7)	7 (0.6)		
	18.5 to <25	235 (31.0)	476 (39.6)	[Reference]	[Reference]
	25 to <30	351 (46.2)	542 (45.1)	1.32 [1.07-1.64]	1.28 [1.02-1.60]
	≥30	168 (22.1)	178 (14.8)	1.91 [1.46-2.49]	1.82 [1.36-2.43]
	Per 5-unit increase			1.33 [1.18-1.49]	1.33 [1.17-1.50]
	No change[a]	203 (26.7)	404 (33.6)	[Reference]	[Reference]
	Loss ≥2 kg	326 (43.0)	243 (20.2)	2.68 [2.11-3.39]	2.71 [2.10-3.49]
	Gain ≥2 kg	230 (30.3)	556 (46.2)	0.83 [0.66-1.05]	0.85 [0.66-1.08]

[a] Within ± 2 kg.
[b] Adjusted for age and sex.
[c] Adjusted for age, sex, previous lower gastrointestinal endoscopy, CRC family history, education, smoking, alcohol consumption, NSAIDs use, physical activity, and statin use.
Abbreviations: BMI = body mass index, CRC = colorectal cancer, NSAIDs = nonsteroidal anti-inflammatory drugs, OR = odds ratio.

© 2023 Mandic M et al. JAMA Network Open.

eTable 6. Rectum - risk according to BMI and weight change at various intervals before diagnosis/interview

Time window	BMI category / weight change	Cases No (%)	Controls No (%)	OR (95% CI)	
				Model 1[b]	Model 2[c]
Diagnosis /interview	<18.5	53 (2.1)	29 (0.5)	3.98 [2.53-6.40]	3.82 [2.27-6.53]
	18.5 to <25	939 (37.5)	1823 (33.4)	[Reference]	[Reference]
	25 to <30	1054 (42.1)	2526 (46.3)	0.79 [0.70-0.88]	0.77 [0.68-0.87]
	≥30	456 (18.2)	1075 (19.7)	0.80 [0.70-0.92]	0.78 [0.67-0.91]
	Per 5-unit increase			0.85 [0.80-0.90]	0.86 [0.81-0.92]
0-2 years ago	<18.5	10 (2.0)	7 (0.6)		
	18.5 to <25	155 (31.1)	401 (33.3)	[Reference]	[Reference]
	25 to <30	219 (44.0)	562 (46.7)	0.98 [0.77-1.26]	1.04 [0.79-1.37]
	≥30	114 (22.9)	233 (19.4)	1.26 [0.94-1.68]	1.30 [0.94-1.81]
	Per 5-unit increase			1.08 [0.96-1.22]	1.12 [0.98-1.28]
	No change[a]	297 (59.6)	1045 (86.9)	[Reference]	[Reference]
	Loss ≥2 kg	164 (32.9)	76 (6.3)	7.42 [5.48-10.05]	9.33 [6.47-13.47]
	Gain ≥2 kg	37 (7.4)	82 (6.8)	1.58 [1.05-2.38]	1.55 [0.98-2.46]
2-4 years ago	<18.5	6 (1.2)	3 (0.3)		
	18.5 to <25	136 (26.9)	330 (31.7)	[Reference]	[Reference]
	25 to <30	239 (47.3)	499 (47.9)	1.17 [0.90-1.51]	1.17 [0.87-1.57]
	≥30	124 (24.6)	209 (20.1)	1.41 [1.04-1.91]	1.49 [1.05-2.12]
	Per 5-unit increase			1.11 [0.98-1.26]	1.16 [1.00-1.34]
	No change[a]	192 (38.0)	665 (63.9)	[Reference]	[Reference]
	Loss ≥2 kg	184 (50.5)	184 (17.7)	4.71 [3.66-6.05]	4.74 [3.54-6.34]
	Gain ≥2 kg	58 (11.5)	192 (18.4)	0.97 [0.69-1.36]	1.03 [0.71-1.50]
4-6 years ago	<18.5	3 (0.6)	8 (0.8)		
	18.5 to <25	158 (29.4)	349 (33.7)	[Reference]	[Reference]
	25 to <30	236 (43.9)	483 (46.6)	1.06 [0.83-1.36]	1.03 [0.78-1.37]
	≥30	141 (26.2)	196 (18.9)	1.57 [1.18-2.10]	1.35 [0.97-1.88]
	Per 5-unit increase			1.25 [1.11-1.41]	1.20 [1.04-1.37]
	No change[a]	178 (33.1)	556 (53.7)	[Reference]	[Reference]
	Loss ≥2 kg	285 (53.0)	215 (20.8)	4.10 [3.20-5.24]	4.34 [3.27-5.76]
	Gain ≥2 kg	75 (13.9)	265 (25.6)	0.83 [0.61-1.13]	0.76 [0.54-1.08]
6-8 years ago	<18.5	3 (0.6)	7 (0.6)		
	18.5 to <25	138 (28.9)	371 (33.4)	[Reference]	[Reference]
	25 to <30	228 (47.7)	515 (46.4)	1.19 [0.92-1.54]	1.16 [0.87-1.54]
	≥30	109 (22.8)	217 (19.5)	1.34 [0.99-1.82]	1.32 [0.94-1.87]
	Per 5-unit increase			1.17 [1.04-1.32]	1.19 [1.04-1.37]
	No change[a]	161 (33.7)	544 (49.0)	[Reference]	[Reference]
	Loss ≥2 kg	220 (46.0)	237 (21.4)	3.17 [2.45-4.10]	3.22 [2.41-4.30]
	Gain ≥2 kg	97 (20.3)	329 (29.6)	0.93 [0.69-1.24]	0.83 [0.61-1.14]
8-10 years ago	<18.5	1 (0.2)	6 (0.6)		
	18.5 to <25	141 (29.2)	401 (37.7)	[Reference]	[Reference]
	25 to <30	223 (46.2)	479 (45.1)	1.33 [1.03-1.71]	1.25 [0.93-1.67]
	≥30	118 (24.4)	177 (16.7)	1.92 [1.41-2.61]	2.12 [1.49-3.03]
	Per 5-unit increase			1.26 [1.11-1.42]	1.32 [1.14-1.52]
	No change[a]	142 (29.4)	474 (44.6)	[Reference]	[Reference]
	Loss ≥2 kg	245 (50.7)	215 (20.2)	3.91 [3.00-5.10]	3.49 [2.59-4.70]
	Gain ≥2 kg	96 (19.9)	374 (35.2)	0.79 [0.59-1.06]	0.72 [0.52-1.00]
10-12 years ago	<18.5	6 (1.2)	7 (0.6)		
	18.5 to <25	150 (30.1)	476 (39.6)	[Reference]	[Reference]
	25 to <30	231 (46.4)	542 (45.1)	1.38 [1.08-1.77]	1.47 [1.12-1.94]
	≥30	111 (22.3)	178 (14.8)	2.06 [1.52-2.80]	2.23 [1.57-3.15]
	Per 5-unit increase			1.30 [1.15-1.48]	1.35 [1.17-1.56]
	No change[a]	118 (23.7)	404 (33.6)	[Reference]	[Reference]
	Loss ≥2 kg	236 (47.4)	243 (20.2)	3.41 [2.59-4.48]	3.41 [2.51-4.64]
	Gain ≥2 kg	144 (28.9)	556 (46.2)	0.81 [0.61-1.08]	0.79 [0.58-1.07]

[a] Within ± 2 kg.
[b] Adjusted for age and sex.
[c] Adjusted for age, sex, previous lower gastrointestinal endoscopy, CRC family history, education, smoking, alcohol consumption, NSAIDs use, physical activity, and statin use.
Abbreviations: BMI = body mass index, CRC = colorectal cancer, NSAIDs = nonsteroidal anti-inflammatory drugs, OR = odds ratio.

© 2023 Mandic M et al. JAMA Network Open.

eTable 7. CRC risk according to ≥5% weight change s ince different intervals before diagnosis/interview

Time window	Weight change (%)	Cases No (%)	Controls No (%)	OR (95% CI)	
				Model 1[a]	Model 2[b]
**0-2 years ago**	Within ±5	940 (74.8)	1139 (94.7)	[Reference]	[Reference]
	Loss ≥5	262 (20.8)	31 (2.6)	10.32 [6.99-15.24]	11.14 [7.39-16.78]
	Gain ≥5	55 (4.4)	33 (2.7)	1.95 [1.24-3.06]	2.00 [1.22-3.26]
**2-4 years ago**	Within ±5	715 (55.6)	853 (81.9)	[Reference]	[Reference]
	Loss ≥5	489 (38.0)	92 (8.8)	6.30 [4.93-8.06]	5.68 [4.37-7.37]
	Gain ≥5	82 (6.4)	96 (9.2)	1.02 [0.75-1.41]	1.01 [0.64-1.28]
**4-6 years ago**	Within ±5	745 (54.5)	760 (73.4)	[Reference]	[Reference]
	Loss ≥5	494 (36.1)	122 (11.8)	4.12 [3.29-5.15]	3.92 [3.09-4.99]
	Gain ≥5	128 (9.4)	154 (14.9)	0.85 [0.65-1.10]	0.77 [0.58-1.03]
**6-8 years ago**	Within ±5	639 (51.5)	776 (69.9)	[Reference]	[Reference]
	Loss ≥5	450 (36.3)	130 (11.7)	4.19 [3.36-5.23]	3.93 [3.09-4.99]
	Gain ≥5	152 (12.2)	204 (18.4)	0.92 [0.72-1.16]	0.86 [0.66-1.12]
**8-10 years ago**	Within ±5	637 (49.6)	681 (64.1)	[Reference]	[Reference]
	Loss ≥5	458 (35.7)	123 (11.7)	4.00 [3.19-5.02]	3.73 [2.93-4.75]
	Gain ≥5	188 (14.7)	258 (24.3)	0.76 [0.61-0.95]	0.80 [0.63-1.02]
**10-12 years ago**	Within ±5	537 (42.7)	663 (55.1)	[Reference]	[Reference]
	Loss ≥5	432 (34.4)	144 (12.0)	3.71 [2.98-4.62]	3.73 [2.94-4.74]
	Gain ≥5	288 (22.9)	396 (32.9)	0.87 [0.72-1.06]	0.86 [0.70-1.07]

[a] Adjusted for age and sex.

[b] Adjusted for age, sex, previous lower gastrointestinal endoscopy, CRC family history, education, smoking, alcohol consumption, NSAIDs use, physical activity, and statin use.

Abbreviations: BMI = body mass index, CRC = colorectal cancer, NSAIDs = nonsteroidal anti-inflammatory drugs, OR = odds ratio.

© 2023 Mandic M et al. JAMA Network Open.
