# Supplementary material for: Association of Overweight, Obesity, and Recent Weight Loss With Colorectal Cancer Risk
Source: JAMA Netw Open. 2023 Apr 21;6(4):e239556. doi: 10.1001/jamanetworkopen.2023.9556 (PMC10122181; doi:10.1001/jamanetworkopen.2023.9556)
Supplement: Supplement 2. — Data Sharing Statement [file jamanetwopen-e239556-s002.pdf]

Data Sharing Statement

Mandic. Association of Overweight, Obesity, and Recent Weight Loss With Colorectal Cancer Risk. *JAMA Netw Open*. Published April 21, 2023. doi:10.1001/jamanetworkopen.2023.9556

Data
Data available: No
